# Supplementary material for: A method for structure prediction of metal-ligand interfaces of hybrid nanoparticles
Source: Nat Commun. 2019 Sep 3;10:3973. doi: 10.1038/s41467-019-12031-w (PMC6722058; doi:10.1038/s41467-019-12031-w)
Supplement: Supplementary file 1 — Supplementary Information [file 41467_2019_12031_MOESM1_ESM.pdf]

Supplementary Information

A method for structure prediction of metal-ligand interfaces of hybrid nanoparticles

Malola et al.

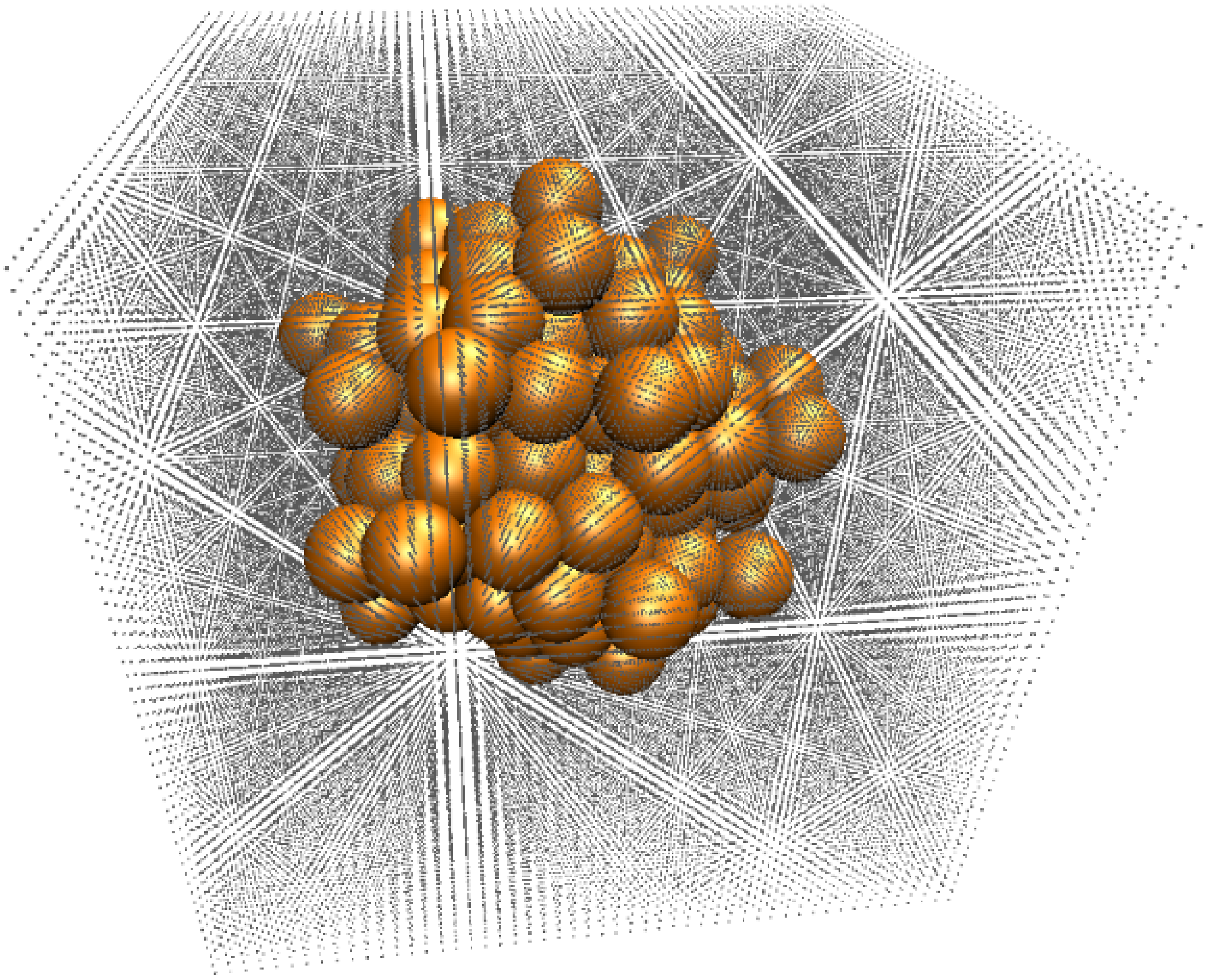

Supplementary Figure 1. Visualization of a 3D grid centered around the gold atoms of a $Au_x(SR)_y$ cluster. Possible sites for sulfur atoms at the gold surface are tested at the grid points using criteria based on the reference structures of known $Au_x(SR)_y$ clusters. In this work, the interval between grid points was 0.2 Å in all directions.

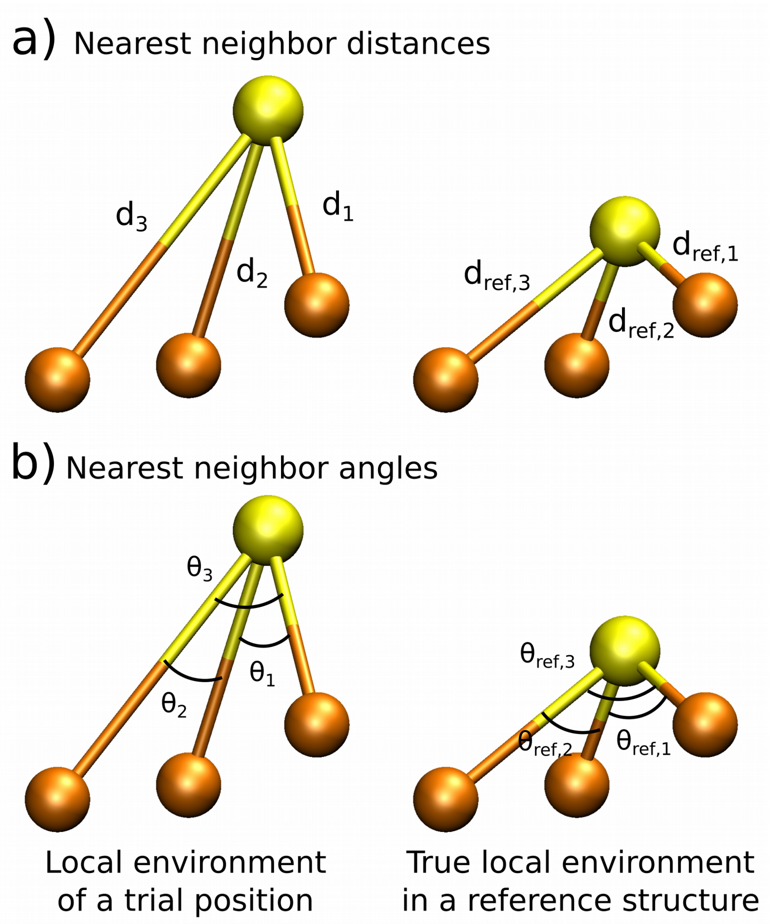

Supplementary Figure 2. An example of three nearest neighbour distances (a) and angles (b) for a trial grid point on the left and for a true S-atom of a reference structure on the right defining the local environment of a S-atom (yellow) with respect to Au-atoms (orange). A match in the selected bond distances and angles is one criterion to accept a grid point (Supplementary Fig.1) as a possible S-atom position. All possible angles between the nearest neighbor atoms to the grid point are tested.

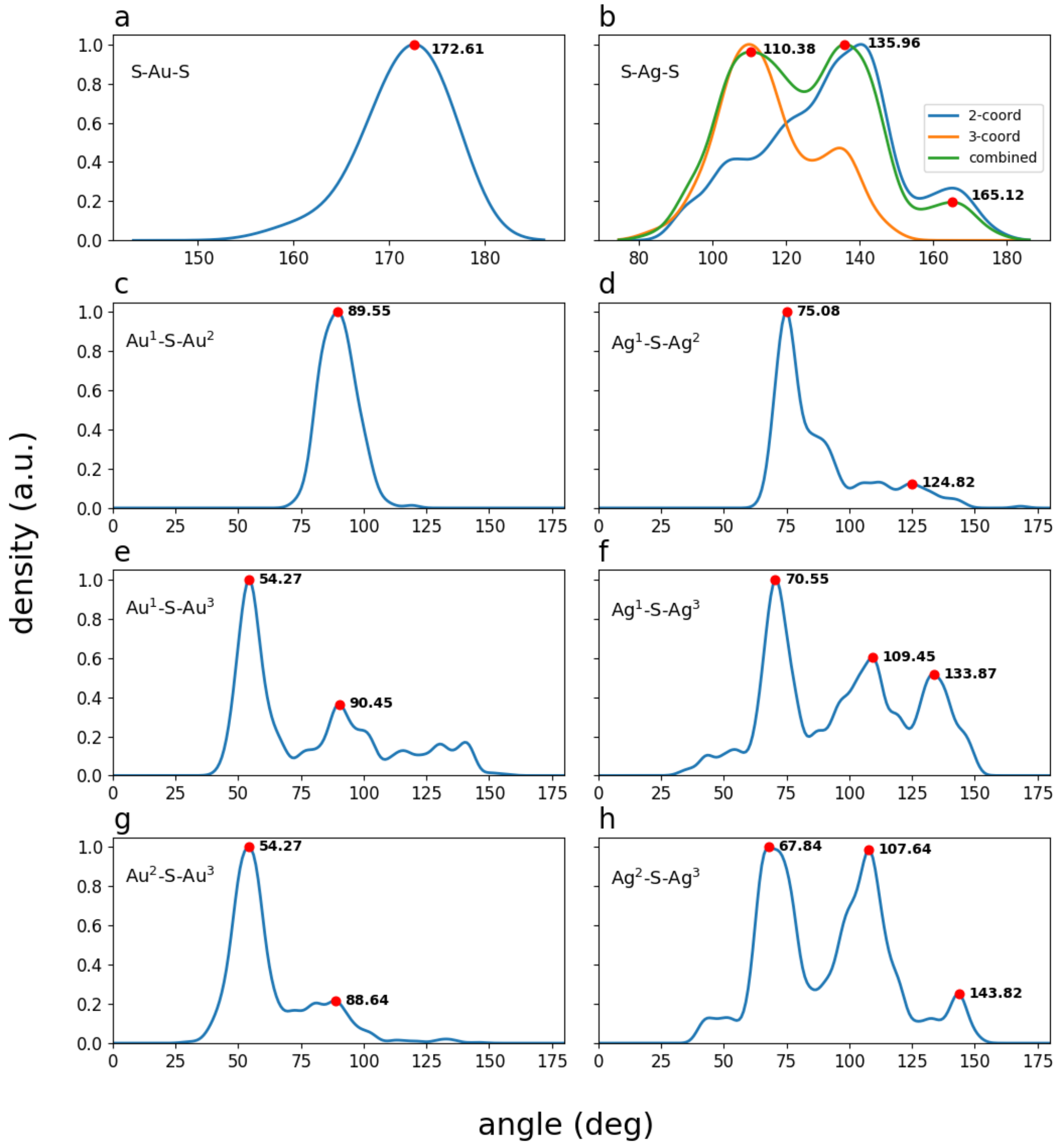

Supplementary Figure 3: Statistics of the nearest neighbor bond angles centered on gold (a), silver (b) and sulfur atoms (c-h) collected the reference structures listed in Supplementary Material. a) The angle formed by S-Au-S configuration is focused close to the 180, ideal in the linear SR-Au-SR conformations. b) the angles formed by S-Ag-S configuration are located close to 180 but also around 120, because of the coordination of silver atom. Major angles are formed tetrahedral-like 3-coordinated silver, triangle-shaped 2-coordinated configuration and almost straight 2-coordinated configuration. c) - h) all nearest neighbor bond angles that can be formed between the sulfur and its three nearest neighbor Au-atoms or Ag-atoms. Superscripts corresponds to the nearest neighbor order. All densities are calculated using gaussian spreading.

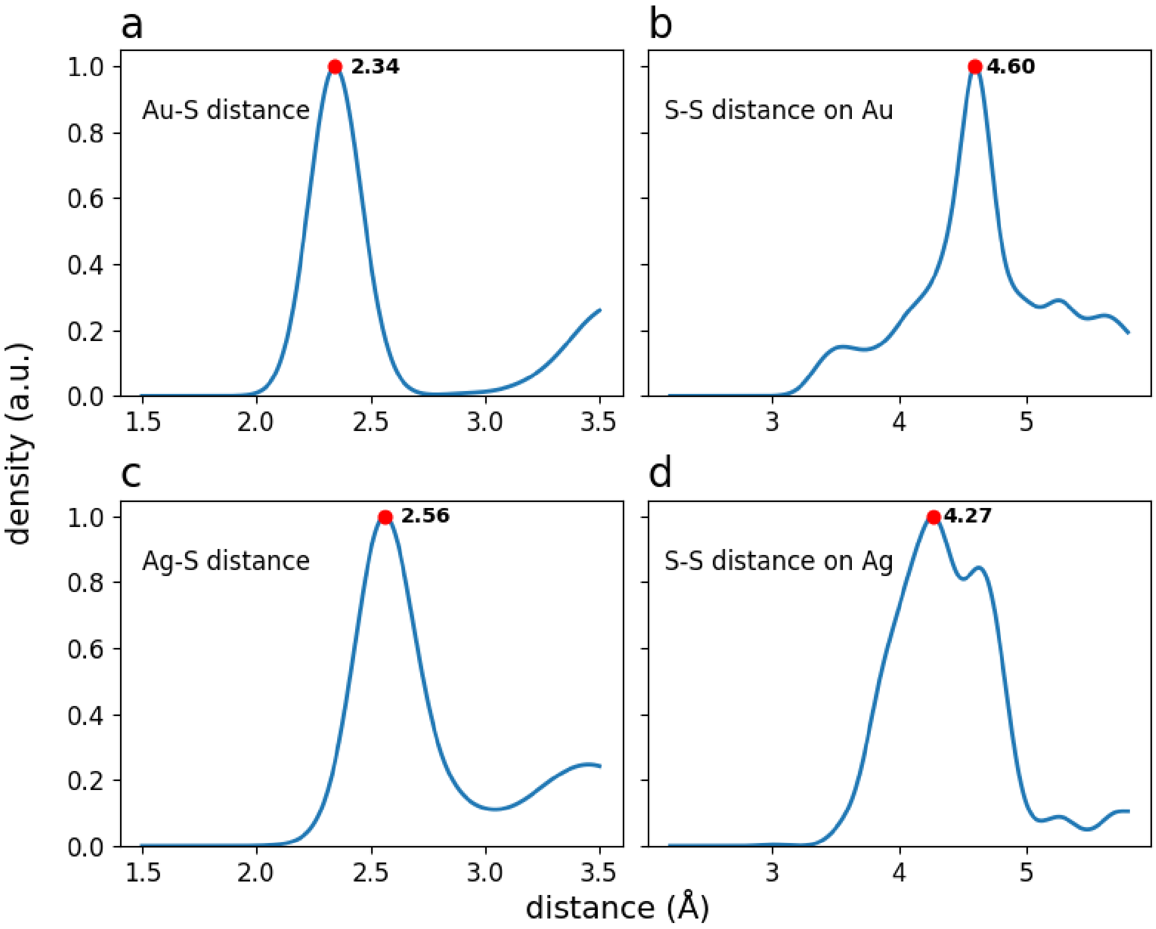

Supplementary Figure 4: Statistics from the atomic distances measured between the metal atoms and the sulfur atoms and between the sulfur atoms from all reference structures in following order: a) distance between gold and sulfur atoms, b) distance between sulfur atoms on gold clusters, c) distance between silver and sulfur atoms, d) distance between sulfur atoms on silver clusters. All densities are calculated using gaussian spreading.

Supplementary Table 1: References for the reference structures of gold clusters. Clusters written with bold text are used in the algorithm as reference structures, but they are not included into the statistics.

Cluster	Reference	Cluster	Reference
$Au_{18}(SC_6H_{11})_{14}$	1, 2	$Au_{38}(PET)_{24}$	17
**$Au_{20}(TBBT)_{16}$**	3	$Au_{42}(SC_6H_{11})_{26}$	14
$Au_{21}(S\text{-}t\text{-}Bu)_{15}$	4	$Au_{44}(TBBT)_{28}$	18
$Au_{21}(S\text{-}Adm)_{15}$	5	$Au_{52}(TBBT)_{32}$	19
$[Au_{23}(SC_6H_{11})_{16}]^-$	6	$Au_{92}(TBBT)_{44}$	20
$Au_{24}(S\text{-}Adm)_{16}$	7	$Au_{102}(p\text{-}MBA)_{44}$	21
$Au_{24}(SCH_2Ph\text{-}t\text{-}Bu)_{20}$	8	$Au_{102}(p\text{-}MBA)_{43}(p\text{-}BBT)$	22
$[Au_{25}(PET)_{18}]^-$	9, 10	$Au_{130}(p\text{-}MBT)_{50}$	23
$Au_{25}(PET)_{18}$	11	$Au_{133}(SPh\text{-}t\text{-}Bu)_{52}$	24
$[Au_{25}(PET)_{18}]^+$	12	**$Au_{144}(SCH_2Ph)_{60}$**	25
$Au_{30}(S\text{-}Adm)_{18}$	13	$Au_{146}(p\text{-}MBA)_{57}$	26
$Au_{34}(SC_6H_{11})_{22}$	14	$Au_{246}(p\text{-}MBT)_{80}$	27
$Au_{36}(SPh\text{-}t\text{-}Bu)_{24}$	15	$Au_{279}(SH)_{84}$	28
$Au_{38}(PET)_{24}$	16		

Supplementary Table 2: References for the reference structures of silver clusters. Clusters written with bold text are used in the algorithm as reference structures, but they are not included into the statistics.

Cluster	Reference	Cluster	Reference
$Ag_{14}(SPhF_2)_{12}(PPh_3)_8$	29	$[Ag_{45}(Dppm)_4(SPh\text{-}t\text{-}Bu)_{16}Br_{12}]^3$	36
$Ag_{16}(DPPE)_4(SPhF_2)_{14}$	30	$Ag_{50}(Dppm)_6(SCH_2Ph\text{-}t\text{-}Bu)_{30}$	37
$Ag_{23}(PPh_3)_8(PET)_{18}$	31	$Ag_{63}(SPhF_2)_{36}(P(C_5H_{11})(C_4H_9)_2)_6(P(C_4H_9)_3)_2$	34
$[Ag_{25}(SPhMe_2)_{18}]^-$	32	**$Ag_{78}(DPPP)_6(SPhCF_3)_{42}$**	38
**$Ag_{29}(BDT)_{12}(PPh_3)_4$**	33	$[Ag_{136}(SPh\text{-}t\text{-}Bu)_{64}Cl_3]^-$	39
$[Ag_{32}(DPPE)_5(SC_6H_4CF_3)_{24}]^{2-}$	30	$[Ag_{141}(S\text{-}Adm)_{40}Br_{12}]^{3+}$	40
$Ag_{38}(SPhF_2)_{26}(P(C_4H_9)_3)_8$	34	$Ag_{210}(SPh\ ^iPr)_{71}(PPh_3)_5$	41
$Ag_{38}(SPhF_2)_{26}(PPh_3)_8$	34	$Ag_{211}(SPh\ ^iPr)_{71}(PPh_3)_6$	41
$[Ag_{44}(SPhF_2)_{30}]^{4-}$	35	$Ag_{374}(SPh\text{-}t\text{-}Bu)_{115}Br_2$	39
$[Ag_{44}(SPhF)_{30}]^{4-}$	35		

Supplementary Table 3: Parameters for predicting thiol positions on the surface of the studied ligand protected Au-clusters.

Cluster	Ligand<br><br>SR / R'	Local environments<br><br>nd / Δd / Δθ	Protecting units<br><br>α1 / Δα	Ligand fitting<br><br>dsph / rsph	Min S to S distances [Å]<br><br>dLL,1 / dLL,2
Au34(SR)22	SC6H11 / C6H11	3 / 0.1 / 10	175 / 5	3.2 / 2.5	3.0 / 3.5
Au36(SR)24	TBBT / Ph	3 / 0.2 / 10	175 / 5	3.2 / 2.5	3.0 / 3.5
Au38(SR)24	PET / CH2	3 / 0.1 / 10	175 / 5	1.8 / 2.1	3.0 / 3.5
Au44(SR)26	2,4-DMBT / DMBT	3 / 0.1 / 10	175 / 5	3.2 / 3.0	3.0 / 3.5
Au52(SR)32	PET / CH2	3 / 0.1 / 10	175 / 5	1.8 / 2.1	3.0 / 3.5
Au92(SR)44	TBBT / Ph	3 / 0.1 / 10	175 / 5	3.2 / 2.5	3.0 / 3.5
Au102(SR)44	pMBA / Ph	3 / 0.2 / 15	175 / 5	3.2 / 2.5	3.0 / 3.5
Au146(SR)57	pMBA / Ph	4 / 0.3 / 20	175 / 5	3.2 / 2.5	3.0 / 3.5
Au279(SR)84	TBBT / Ph	3 / 0.1 / 10	175 / 5	3.2 / 2.5	3.0 / 3.5

Supplementary Table 4: Parameters for predicting sulfur positions on the surface of studied ligand protected Ag-clusters.

Cluster	Ligands<br><br>SR / R'	Local environments<br><br>nd / Δd / Δθ	Protecting units<br><br>α1 / α2 / Δα	Ligand fitting<br><br>dsph / rsph	Min S to S distances [Å]<br><br>dLL,1 / dLL,2
Ag23(SR)18(PPh3)8	PET / CH2	3 / 0.1 / 10	120 / 170 / 5	1.8 / 2.1	3.5 /3.5
Ag44(SR)30	SPhF2 / Ph	4 / 0.2 / 15	120 / 170 / 5	3.2 / 2.5	3.5 /3.5
Ag78(SR)42(DPPP)6	SPhCF3 / Ph	3 / 0.1 / 10	120 / 170 / 5	3.2 / 2.5	3.5 /3.5
Ag211Cl(SR)71(PPh3)6	SPhiPr / Ph	3 / 0.1 / 10	120 / 170 / 5	3.2 / 2.5	3.5 /3.5

Supplementary Table 5: Parameters for predicting phosphine and diphosphine positions for ligand protected Ag-clusters. Predictions are made starting with a initial model structure after the prediction of sulfur positions (see in Tables 1 and 2).

Cluster	Phosphine ligand	Local environments<br><br>nd / Δd / Δθ	P to P distances in diphosphines<br><br>ddiP	Ligand fitting<br><br>dsph / rsph	Min P to P distances [Å]<br><br>dLL,1 / dLL,2
Ag23(SR)18(PPh3)8	PPh3	4 / 0.1 / 10	-	3.4 / 4.6	- / 3.5
Ag78(SR)42(DPPP)6	DPPP	4 / 0.2 / 10	5.0 – 5.5	3.4 / 4.6	3.5 / 3.5
Ag211Cl(SR)71(PPh3)6	PPh3	4 / 0.2 / 10	-	3.4 / 4.6	- / 3.5

Supplementary Table 6. The results of predicting phosphine and diphosphine positions (PPh_3 or DPPP) for ligand protected Ag-clusters.

Cluster	Number of model structures	Max in tot	Correct structures %<br><br>4/5 of nn correct for each atom	Min RMSD of correct structures [Å]
$Ag_{23}(SR)_{18}(PPh_3)_8$	3072	8	100% (3072)	0.266
$Ag_{78}(SR)_{42}(DPPP)_6$	3072	6	100% (3072)	0.480
$Ag_{211}Cl(SR)_{71}(PPh3)_6$	3072	6	100% (3072)	0.633

Supplementary Table 7: Parameters of predicting sulfur atom positions Au44(SR)26 and Au44(SR)28 using different size of spherical probes for ligands.

Cluster	Ligand<br><br>SR	Local environments<br><br>nd / Δd / Δθ	Protecting units<br><br>α1 / Δα	Ligand fitting<br><br>dsph / rsph	Min S to S distances [Å]<br><br>dLL,1 / dLL,2
Au44(SR)26	2,4-DMBT	3 / 0.1 / 10	175 / 5	3.2 / 3.0	3.0 / 3.5
Au44(SR)28	TBBT	3 / 0.2 / 10	175 / 5	3.2 / 2.8	3.0 / 3.5
Au44(SR)28	TBBT	3 / 0.1 / 10	175 / 5	3.2 / 3.0	3.0 / 3.5

Supplementary Table 8. The results of predicting sulfur positions for $Au_{44}(SR)_{28}$ using different size of spherical probes for ligand fitting as compared to $Au_{44}(SR)_{26}$ cluster.

Cluster	Number of model structures	Ligand<br><br>SR	Ligand fitting<br><br>dsph / rsph	Max in units	In total if max in units	Correct structures in %<br><br>2/2 of nn correct for each atom
$Au_{44}(SR)_{26}$	9216	2,4-DMBT	3.2 / 3.0	24	25-26	28.0 % (2579)
$Au_{44}(SR)_{28}$	9216	TBBT	3.2 / 2.8	28	28	5.90 % (544)
$Au_{44}(SR)_{28}$	9216	TBBT	3.2 / 3.0	24	26	0.00 % (0)

Supplementary Note 1: Algorithms 1-4

Algorithm 1 Create a model structure

Require: Database of reference structures (training set)
Ensure: Set of model structures for the metal-ligand interface

```
Define coordinates of metal atoms
Find possible interface atom positions around the metal atoms, using information about
    the local chemical environments in the training set
for N times do
    Generate a random, plausible model structure
Rank each model structure based on selected criteria, and select the best one(s)
```

Algorithm 2 Generate a random, plausible model structure

Require: Coordinates of metal atoms G_{M0}, initial plausible interface atom positions G_{S0}, minimum distance of isolated interface atoms $d_{LL,2}$
Ensure: a random plausible model structure, final coordinates of interface atoms G_{Sf}

$G_{Si} \leftarrow G_{S0}$
$G_{Sf} \leftarrow \emptyset$
while $G_{Si} \neq \emptyset$ **do**
    $p_{i1} \leftarrow random(G_{Si})$
    $(G_{Si}, G_{Sf}) \leftarrow TryLocalStruct(p_{i1}, G_{Si}, G_{Sf}, G_{M0})$
    $G_{Si} \leftarrow RemoveImpossible(G_{Si}, G_{Sf}, G_{M0})$
    $G_{Si} \leftarrow \{p_j : p_j \in G_{si}; p_j \neq p_{i1}\}$
$G_{Si} \leftarrow G_{S0}$
for $p_f \in G_{Sf}$ **do**
    $G_{Si} \leftarrow G_{Si} \setminus \{p_j : p_j \in G_{Si}; d(p_j, p_f) < d_{LL,2}\}$
$G_{Si} \leftarrow RemoveImpossible(G_{Si}, G_{Sf}, G_{M0})$
while $G_{Si} \neq \emptyset$ **do**
    $p_i \leftarrow random(G_{Si})$
    $G_{Sf} \leftarrow G_{Sf} \cup \{p_i\}$
    $G_{Si} \leftarrow G_{Si} \setminus (\{p_i\} \cup \{p_j : p_j \in G_{Si}; d(p_j, p_i) < d_{LL,2}\})$
    $G_{Si} \leftarrow RemoveImpossible(G_{Si}, G_{Sf}, G_{M0})$

Algorithm 3 *TryLocalStruct*$(p_{i1}, G_{Si}, G_{Sf}, G_{M0})$ //
Add specified local structure if possible and remove positions based on distances

Require: Trial interface atom coordinate $p_{i1} \in G_{Si}$, initial plausible interface atom positions G_{Si}, final selected interface atom positions G_{Sf}, coordinates of metal atoms G_{M0}, minimum distance of interface atoms of specified local structure $d_{LL,1}$, possible bond angles α_i and error $\Delta\alpha_i$ between two interface atoms and the nearest metal atom in the local structure.

Ensure: Updated initial plausible interface atom positions G_{Si}, updated final coordinates of interface atoms G_{Sf}

for $p_M \in \{p_{Mj} : p_{Mj} \in G_{M0}; d(p_{Mj}, p_{i1}) \in [1.95\text{Å}, 2.75\text{Å}]\}$ **do**
    $G_1 \leftarrow \{p_j : p_j \in G_{Sf}; d(p_j, p_M) \in [1.95\text{Å}, 2.75\text{Å}] \bigwedge \theta(p_{i1}, p_M, p_j) \in [\alpha_i - \Delta\alpha_i, \alpha_i + \Delta\alpha_i]\}$
    if $G_1 \neq \emptyset$ **then**
        $G_{Sf} \leftarrow G_{Sf} \cup \{p_{i1}\}$
        $G_{Si} \leftarrow G_{Si} \setminus (\{p_{i1}\} \cup \{p_j : p_j \in G_{Si}; d(p_j, p_{i1}) < d_{LL,1}\})$
        break
    else
        $G_2 \leftarrow \{p_j : p_j \in G_{Si}; d(p_j, p_M) \in [1.95, 2.75] \bigwedge \theta(p_{i1}, p_M, p_j) \in [\alpha_i - \Delta\alpha_i, \alpha_i + \Delta\alpha_i]\}$
        if $G_2 \neq \emptyset$ **then**
            $p_{i2} \leftarrow SelectFirst(G_2)$ // (first member of the set)
            $G_{Sf} \leftarrow G_{Sf} \cup \{p_{i1}, p_{i2}\}$
            $G_{Si} \leftarrow G_{Si} \setminus (\{p_{i1}, p_{i2}\} \cup \{p_j : p_j \in G_{Si}; d(p_j, p_{i1}) < d_{LL,1} \bigvee d(p_j, p_{i2}) < d_{LL,1}\})$
            break

Algorithm 4 *RemoveImpossible*(G_{Si}, G_{Sf}, G_{M0}) //
Remove initial positions based on metal atom coordination

Require: Initial plausible interface atom positions G_{Si}, final selected interface atom positions G_{Sf}, coordinates of metal atoms G_{M0}, maximum coordination of metal atoms $cnumb_{max}$

Ensure: Updated initial plausible S-atom positions G_{Si}

for $p_M \in G_{M0}$ **do**
    $cnumb \leftarrow size(\{p_f : p_f \in G_{Sf}; d(p_f, p_M) < 2.75\text{Å}\})$
    if $cnumb \geq cnumb_{max}$ **then**
        $G_{Si} \leftarrow G_{Si} \setminus \{p_j : p_j \in G_{Si}; d(p_j, p_M) < 2.75\text{Å}\}$

Supplementary Note 2: Validation of selected parameters.

The algorithm is based on a restricted structural search in which the simple rules depict the crucial physical and chemical insights into the process. The reference structures define the chemistry in form of local environments of atoms when finding the plausible interface atom positions (Algorithm 2). The realistic interface atom positions are accepted based on the nearest neighbor bond distances and bond angles within prescribed error limits. In the algorithm these are parameterized and in this study 3-4 nearest neighbors with 0.1-0.3 Å error limits for individual bond distances and 10-20 degrees for individual bond angles is used. The selected error limits of the bond distances and bond angles reflect the statistics collected from the known reference structures of the same class as listed in the Supplementary Material. We analysed statistics from the nearest neighbor Au-S, Ag-S bond distances and from Au-S-Au and Ag-S-Ag bond angles which are shown in Supplementary Figs. 3 and 4. These parameters dictate how close the local environment of a trial position has to be to the known environments of reference structures in order to be accepted. As a general instruction for prediction of more complex systems, it is better to increase the number of nearest neighbors and loosen the error limits for the bonds and angles instead of decreasing the number of nearest neighbors and restricting the criteria. The same instruction is valid when the number of reference structures is low.

During the search of realistic interface atom positions in Algorithm 2, spatial fitting of the ligands is made with spherical probe in perpendicular direction to the two nearest neighbor Au-S and Ag-S bonds for thiolates and parallel to the nearest neighbor Ag-P bond for phosphines (see Figure 2), which are the most natural bonding directions for the organic part of ligands in these systems. In addition to the bonding direction, the representative spherical probes have to be parameterized for the size and for the distance. The spherical probe should mimic the most important, rigid, molecular organic group closest to the binding site atom. In our study all the parameters are estimated based on the atomic distances.

From Supplementary Fig. 4 we can see that the nearest neighbor bond distributions have single maxima at 2.3-2.5 Å for both Au-S and Ag-S bonds. Broadening of the distribution confirms that all the nearest neighbor metal atoms of sulfur in protected Au- and Ag-clusters can be found within 1.95 – 2.75 Å, which is one the guiding lines used in structural search when nearest neighbor metal and interface atoms are defined in both Au- and Ag-clusters. Determining the covalently bound metal and interface atoms is important during the restricted model structure generation. By looking at the distributions of S-Au-S and S-Ag-S bond angles in Supplementary Fig. 3 it is realized that there exist some favorable local arrangements of metal and interface atoms. When model structures are generated, it is reasonable to try adding first the most favorable conformations for which Algorithm 3 concentrates on. In the protected Au-clusters, the linear SR-Au-SR conformations that form different length of SR-(Au-SR)n (N=1,2) protecting units, are in the majority. In protected Ag-clusters, the coordination of Ag-atoms to

S-atoms is more flexible including also three-coordinated arrangements in addition to two-coordinated linear SR-Ag-SR conformations. From the analysis shown in Supplementary Fig. 3 we can see that the linear conformations create a maxima in Au-S-Au and Ag-S-Ag bond angles close to 180 degrees whereas for Ag-clusters there is lot of weight on angles around 120 degrees. These observations led us to define special local conformations based on relative angles between metal and interface atoms. In the first part, adding interface atoms only into such specific local structures will be allowed that have S-Au-S angles of 175 degrees and S-Ag-S angles of 120 and 170 degrees. At the same time, the maximum coordination of metal atoms to sulfur can be restricted to 2 for Au- and 3 for Ag-atoms (see Algorithm 4). For gold atoms, no three-coordinated conformations to sulfur are seen in the known thiolate protected clusters. For phosphines, the only special local structure that limits the prediction is related to diphosphines for which the distance between the individual P atoms is restricted by the length of the connecting organic molecular group. Hence, the P atoms are added in pairs within a restricted distance range.

During generation of the model structure it is important that the distances between the added interface atoms remain reasonable. Based on the analysis of the S to S distances on the surface of the known protected Au-clusters shown in Supplementary Fig. 4 we can see that the lower edge of the distance distribution is located at 3.0 Å in protected Au-clusters and at 3.5 Å in protected Ag-clusters. Because of that we restrict the minimum distance between added interface atoms to be at 3.0 Å for the S-atoms in linear S-Au-S conformations and at 3.5 Å for the bridged thiolates on Au-S interfaces. For Ag-S and Ag-P interfaces a minimum distance of 3.5 Å is used for all added interface atoms in all possible conformations. In true system ligands that are closer to the given limits have high probability to form a dimer and become detached from the surface in ambient conditions, hence, no S-S bonds are seen on the surface of the known protected Au- and Ag-clusters.

Supplementary Note 3: Combined structural error (CSE)

CSE is calculated from four different contributions: bond distances, bond angles, number of interface atoms in special local conformations, number of interface atoms in total. The error is calculated from all contributions as a relative number (percentages from the expected values) without special units.

$$\text{CSE} = \frac{1}{4}\left[\Delta nn + \Delta N_{unit} + \Delta N_{tot}\right] \tag{1}$$

$$\Delta N_{unit} = \frac{\left|N_{unit} - N_{max,unit}\right|}{N_{max,unit}} \tag{2}$$

$$\Delta N_{tot} = \frac{\left|N_{tot} - N_{max,tot}\right|}{N_{max,tot}} \tag{3}$$

where $N_{max,tot}$ is defined from the set of generated model structures having maximum number, $N_{max,unit}$, of interface atoms in special local structure conformations (like protecting units). N_{unit} and N_{tot} are the number of interface atoms in special local conformations and in total respectively for the model structure in question.

$$\Delta nn = \frac{1}{N}\sum_{i=1}^{N} min_j\left[\Delta d_{ij} + \Delta\theta_{ij}\right] \tag{4}$$

$$\Delta d_{ij} = \frac{1}{n_d}\sum_{m=1}^{n_d} \frac{\left|d_m^i - d_m^{j,ref}\right|}{d_m^{j,ref}} \tag{5}$$

$$\Delta\theta_{ij} = \frac{1}{n_\theta}\sum_{m=1}^{n_\theta} \frac{\left|\theta_m^i - \theta_m^{j,ref}\right|}{\theta_m^{j,ref}} \tag{6}$$

where d_m^i and θ_m^i refer to *m*th nn-bond distance and angle for a model structure atom i and $d_m^{j,ref}$ and $\theta_m^{j,ref}$ to *m*th nn-bond distance and angle for a reference structure atom j. For all atoms the minimum average error in local environment of n_d nearest neighbor distances and n_θ nearest neighbor angles contributes in the combined structural error.

Minimization of the error contributions is done with respect to the known local environments of similar type of atoms j found in the set of reference structures.

Supplementary Note 4: Approach for optimizing the process against stochastic challenges.

To overcome the challenges of the non-guided stochastic process, we introduce one possible approach to optimize the structural search. In more complex systems with increasing number of possible metal-ligand interface conformations the structural search can be improved by repeating the algorithm multiple times. For each repetition, the set of possible interface atom positions should be constructed from interface atoms of the best model structures of previous run, ranked by criteria discussed later. In this study, we have used 20 best model structures for this purpose, which have the largest number of interface atoms in specified protecting motifs and in total.

References

1. Das A. et al. Structure determination of [Au18(SR)14]. *Angew. Chemie* **54**, 3140-3144 (2015)
2. Chen, S. et al. The structure and optical properties of the [Au18(SR)14] nanocluster. *Angew. Chemie* **54**, 3145-3149 (2015)
3. Zeng, C., Chong, L., Chen, Y., Rosi, N.L. & Jin, R. Gold–thiolate ring as a protecting motif in the Au20(SR)16 nanocluster and implications. *J. Am. Chem. Soc* **136***,* 11922-11925 (2014)
4. Yang, S. et al. In situ two-phase ligand exchange: a new method for the synthesis of alloy nanoclusters with precise atomic structures. *J. Am. Chem. Soc.* **139**, 5668-5671 (2017)
5. Chen, S. et al. Total structure determination of Au21(S-Adm)15 and geometrical/electronic structure evolution of thiolated gold nanoclusters. *J. Am. Chem. Soc.* **138**, 10754-10757 (2016)
6. Das, A. et al. Nonsuperatomic [Au23(SC6H11)16]− nanocluster featuring bipyramidal Au15 kernel and trimeric Au3(SR)4 motif. *J. Am. Chem. Soc.* **135**, 18264-18267 (2013)
7. Crasto, D. et al. Au24(SAdm)16 nanomolecules: X-ray crystal structure, theoretical analysis, adaptability of adamantane ligands to form Au23(SAdm)16 and Au25(SAdm)16, and its relation to Au25(SR)18. *J. Am. Chem. Soc.* **136**, 14933-14940 (2014)
8. Das, A. et al. Crystal structure and electronic properties of a thiolate-protected Au24 nanocluster. *Nanoscale* **6**, 6458-6462 (2014)
9. Zhu, M., Aikens, C.M., Hollander, F.J., Schatz, G.C. & Jin, R. Correlating the crystal structure of a thiol-protected Au25 cluster and optical properties. *J. Am. Chem. Soc.* **130**, 5883-5885 (2008)
10. Heaven, M.W., Dass, A., White, P.S., Holt, K.M. & Murray, R.W. Crystal structure of the gold nanoparticle [N(C8H17)4][Au25(SCH2CH2Ph)18]. *J. Am. Chem. Soc.* **130**, 3754-3755 (2008)

11. Zhu, M., Eckenhoff, W.T., Pintauer, T. & Jin, R. Conversion of anionic [Au25(SCH2CH2Ph)18]− cluster to charge neutral cluster via air oxidation. *J. Phys. Chem. C* **112**, 14221-14224 (2008)
12. Tofanelli, M.A. et al. Jahn–Teller effects in Au25(SR)18. *Chem. Sci.* **7**, 1882-1890 (2016)
13. Higaki, T. et al. Controlling the atomic structure of Au30 sanoclusters by a ligand−based strategy. *Angew. Chemie* **55**, 6694-6697 (2016)
14. Dong, H. et al. A novel double-helical-kernel evolution pattern of gold nanoclusters: alternate single-stranded growth at both ends. *Nanoscale* **9**, 3742-3746 (2017)
15. Zeng, C. et al. Total structure and electronic properties of the gold nanocrystal Au36(SR)24. *Angew. Chemie* **51**, 13114-13118 (2012)
16. Qian, H., Eckenhoff, W.T., Zhu, Y., Pintauer, T. & Jin, R. Total structure determination of thiolate-protected Au38 sanoparticles, *J. Am. Chem. Soc.* **132**, 8280-8281 (2010)
17. Tian, S. et al. Structural isomerism in gold nanoparticles revealed by X-ray crystallography. *Nat. Commun.* **6**, 8667 (2015)
18. Zeng, C. et al. Gold quantum boxes: on the periodicities and the quantum confinement in the Au28, Au36, Au44, and Au52 magic series. *J. Am. Chem. Soc.* **138**, 3950-3953 (2016)
19. Zhuang, S. et al. The fcc structure isomerization in gold nanoclusters. *Nanoscale* **9**, 14809-14813 (2017)
20. Zeng, C., Liu, C., Chen, Y., Rosi, N.L. & Jin, R. Atomic structure of self-assembled monolayer of thiolates on a tetragonal Au92 nanocrystal. *J. Am. Chem. Soc.* **138**, 8710-8713 (2016)
21. Jadzinsky, P.D., Calero, G., Ackerson, C.J., Bushnell, D.A. & Kornberg, R.D. Structure of a thiol monolayer-protected gold nanoparticle at 1.1 Å Resolution. *Science* **318**, 430-433 (2007)
22. Heinecke, C.L. et al. Structural and theoretical basis for ligand exchange on thiolate monolayer protected gold nanoclusters. *J. Am. Chem. Soc.* **134**, 13316-13322 (2012)
23. Chen, Y. et al. Crystal structure of barrel-shaped chiral Au130(p-MBT)50 nanocluster. *J. Am. Chem. Soc.* **137**, 10076-10079 (2015)
24. Zeng, C. et al. Structural patterns at all scales in a nonmetallic chiral Au133(SR)52 nanoparticle. *Sci. Adv.* **1**, e1500045 (2015)
25. Yan, N. et al. Unraveling the long-pursued Au144 structure by X-ray crystallography, *Sci. Adv.* **4**, eaat7259 (2018)
26. Vergara, S. et al. MicroED structure of Au146(p-MBA)57 at subatomic resolution reveals a twinned FCC cluster. *J. Phys. Chem. Lett.* **8**, 5523-5530 (2017)
27. Zeng, C., Chen, Y., Kirschbaum, K., Lambright, K.J. & Jin, R., Emergence of hierarchical structural complexities in nanoparticles and their assembly, *Science* **354**, 1580-1584 (2016)
28. Sakthivel, N.A., Theivendran, S., Ganeshraj, V., Oliver, A.G. & Dass, A. Crystal structure of faradaurate-279: Au279(SPh-tBu)84 plasmonic nanocrystal molecules. *J. Am. Chem. Soc.* **139**, 15450-15459 (2017)
29. Yang, H. et al. Crystal structure of a luminescent thiolated Ag nanocluster with an octahedral Ag64+ core. *Chem. Commun.* **49**, 300-302 (2013)
30. Yang, H., Wang, Y. & Zheng, N. Stabilizing subnanometer Ag(0) nanoclusters by thiolate and diphosphine ligands and their crystal structures. *Nanoscale* **5**, 2674-2677 (2013)

31. Liu, C. et al. Chiral Ag23 nanocluster with open shell electronic structure and helical face-centered cubic framework. *Nat. Commun.* **9**, 744 (2018)
32. Joshi, C.P., Bootharaju, M.S., Alhilaly, M.J. & Bakr, O.M. [Ag25(SR)18]−: the “golden” silver nanoparticle. *J. Am. Chem. Soc.* **137**, 11578-11581 (2015)
33. AbdulHalim, L.G. et al. Ag29(BDT)12(TPP)4: a tetravalent nanocluster. *J. Am. Chem. Soc.* **137**, 11970-11975 (2015)
34. Yang, H. et al. Embryonic growth of face-center-cubic silver nanoclusters shaped in nearly perfect half-cubes and cubes. *J. Am. Chem. Soc.* **139**, 31-34 (2017)
35. Yang, H. et al. All-thiol-stabilized Ag44 and Au12Ag32 nanoparticles with single-crystal structures, *Nat. Commun.* **4**, 2422 (2013)
36. Zou, X. et al. Multi-ligand-directed synthesis of chiral silver nanoclusters. *Nanoscale* **9**, 16800-16805 (2017)
37. Du, W. et al. Ag50(Dppm)6(SR)30 and its homologue AuxAg50–x(Dppm)6(SR)30 alloy nanocluster: seeded growth, structure determination, and differences in properties. *J. Am. Chem. Soc.* **139**, 1618-1624 (2017)
38. Yang, H. et al. From racemic metal nanoparticles to optically pure enantiomers in one pot. *J. Am. Chem. Soc.* **139**, 16113-16116 (2017)
39. Yang, H. et al. Plasmonic twinned silver nanoparticles with molecular precision. *Nat. Commun.* **7**, 12809 (2016)
40. Ren, L. et al. Bulky surface ligands promote surface reactivities of [Ag141X12(S-Adm)40]3+ (X = Cl, Br, I) nanoclusters: models for multiple-twinned nanoparticles, *J. Am. Chem. Soc.* **139**, 13288-13291 (2017)
41. Liu, J. et al. Different silver nanoparticles in one crystal: Ag210(iPrPhS)71(Ph3P)5Cl and Ag211(iPrPhS)71(Ph3P)6Cl, *Angew. Chemie* **58**, 195-199 (2019)
